# Senescence Marker Protein 30 (SMP30): A Novel Pan-Species Diagnostic Marker for the Histopathological Diagnosis of Breast Cancer in Humans and Animals

**DOI:** 10.3390/ijms22052340

**Published:** 2021-02-26

**Authors:** Su-Min Baek, Seoung-Woo Lee, Tae-Un Kim, Seong-Kyoon Choi, Sungho Yun, Won-Jae Lee, Se-Hyeon Han, Il-Hwa Hong, Sang-Joon Park, Tae-Hwan Kim, Kyu-Shik Jeong, Jin-Kyu Park

**Affiliations:** 1 Department of Veterinary Pathology, College of Veterinary Medicine, Kyungpook National University, Daegu 41566, Korea; suminbaek@naver.com (S.-M.B.); pyrk2000@gmail.com (S.-W.L.); tukim92@naver.com (T.-U.K.); vetman@sbs.co.kr (S.-H.H.); thkim56@knu.ac.kr (T.-H.K.); jeongks@knu.ac.kr (K.-S.J.); 2 Core Protein Resources Center, Daegu Gyeongbuk Institute of Science and Technology (DGIST), Daegu 42988, Korea; cskbest@dgist.ac.kr; 3 College of Veterinary Medicine, Kyungpook National University, Daegu 41566, Korea; shyun@knu.ac.kr (S.Y.); iamcyshd@knu.ac.kr (W.-J.L.); psj26@knu.ac.kr (S.-J.P.); 4 Department of News Team, Seoul Broadcasting Station (SBS), Seoul 07574, Korea; 5 Department of Veterinary Pathology, College of Veterinary Medicine, Gyeongsang National University, Jinju 52828, Korea; ihhong@gnu.ac.kr; 6 Stem Cell Therapeutic Research Institute, Kyungpook National University, Daegu 41566, Korea

**Keywords:** breast cancer, cat, diagnostic marker, dog, human, mammary carcinoma, neoplastic glandular epithelial cell, SMP30

## Abstract

Senescence marker protein 30 (SMP30) is a cell survival factor playing an important role in vitamin C synthesis and antiapoptosis. Moreover, its cytoprotective role suggests a possibility to be related to cancer cell survival. Mammary carcinoma is a common cancer in both humans and animals. Because of its histopathological diversity, especially in the early stage, histopathological diagnosis may be complicated; therefore, a diagnostic marker is helpful for confirmation. The present study analyzed the expression pattern of SMP30 in mammary carcinoma in humans, dogs, and cats. Immunohistochemistry, immunofluorescence, and western blot analysis were used to investigate SMP30 expression patterns. The expression was specifically observed in neoplastic glandular epithelial cells. The expression increased with the malignancy of glandular epithelial cells with a highly proliferative status. However, SMP30 expression was low in normal mammary gland tissues or well-differentiated adenoma tissues. The patterns were consistently reproduced in canine primary mammary carcinoma cells and MCF-7 and MDA-MB-231 human carcinoma cell lines. This study provides useful information to understand SMP30 expression in various stages of mammary carcinoma and to suggest its utility as a pan-species diagnostic marker, thereby helping to establish strategies for diagnosing mammary carcinoma in several species.

## 1. Introduction

For many years, breast cancer incidence has increased tremendously worldwide. An estimated 627,000 women died of breast cancer, accounting for 15% of cancer deaths in females [1]. An estimated 2.1 million women were diagnosed with breast cancer, which accounts for almost one in four cases of cancer [2]. The incidence and mortality of breast cancer are the highest among all cancers in women and make up 11.6% of all tumors irrespective of sex, which ranks as the second highest [2,3]. Mammary gland tumors are a common type of tumor in animals as well as human beings [4,5]. In particular, in countries where ovariectomy is not frequently performed, the incidence of mammary neoplasia makes up 50% to 70% of all neoplasia in intact female dogs [6]. However, in countries with well-developed medical care services, mammary gland tumors are still reported to be one of the most frequently diagnosed neoplasias. They account for 41.7% of all tumors developed in intact female dogs [7]. Although medical advances have raced ahead in treatment, early diagnosis is still most crucial to improve prognosis and survival rates for mammary gland tumors [8,9].

To date, human epidermal growth factor receptor 2 (HER2), estrogen receptors (ERs), and progesterone receptors (PRs) have been the most well known and routinely used biomarkers for the diagnosis of mammary gland tumors in veterinary fields and humans because they are reliable and strong predictors for the prognosis of endocrine therapy performed in patients with mammary gland tumors [10,11,12,13,14]. However, HER2 is overexpressed in only 10% to 25% of human breast cancers [11]. Although this marker has been studied in various studies, the results were even more variable in canine and feline mammary gland tumors [7]. Moreover, ERs and PRs were not well-established markers in veterinary medicine owing to their great variability of results [15]. Therefore, there was no definitive correlation between expression levels of these markers and prognosis in mammary gland tumors of animals, whereas immunohistochemical observations using ER and PR antibodies were routinely performed in humans as reliable diagnostic and prognostic markers [16]. In other words, there is an absence of reliable diagnostic markers that can be used in both humans and animals with consistent and accurate results. Therefore, there have been continuous attempts to search for the ideal markers with specificity and consistency for diagnosis of mammary gland tumors to help in therapeutic decision making.

Tumor cells are characterized by diminished apoptosis, suppressed cell death, and uncontrolled proliferation [17]. Cancer can be alleviated by promoting tumor cell apoptosis because it is a process of scavenging damaged or surplus cells [18]. Because of dysregulation of cell proliferation, loss of apoptosis results in cancer development [19]. Senescence marker protein 30 (SMP30) is a protein well known for its role in ascorbate biosynthesis in mammals [20]. Recent studies reported that SMP30 is related to many pathophysiological conditions, including cancer [20]. SMP30 is an important cell survival factor for mediating antiapoptotic activity [21,22,23,24,25,26]. SMP30 has been reported to suppress apoptosis through various pathways, including suppression of nitric oxide (NO) synthase, tumor necrosis factor α (TNF- α), lipopolysaccharide (LPS), and the Fas-mediated apoptotic pathway [21,27]. In addition, a few studies reported that this protective role of SMP30 is mediated by the ERK signaling pathway [28,29], which is an important mediator of antiapoptosis, cell proliferation, and cell survival [30]. However, the most important cytoprotective mechanism of SMP30 is the inhibition of reactive oxygen species (ROS)-mediated apoptosis because SMP30 is a key regulator of vitamin C synthesis, and thus, SMP30 is one of the key mediators of antioxidant responses [23,24]. However, this antiapoptotic activity of SMP30 also helps the survival of tumorigenic cells, and repression of SMP30 has a preventative effect in the development of mammary carcinoma [31]. This indicates that SMP30 participates in the proliferation of neoplastic cells. However, the precise role of SMP30 is still controversial. Other studies suggested that the lack of SMP30 exacerbates tumorigenesis [32]. Given the lack of consistency among different researchers, the role and expression pattern of SMP30 in mammary carcinoma should be clarified.

In this study, we investigated the expression patterns of SMP30 in mammary tumors and evaluated its possibility as a pan-species diagnostic marker in canine and feline mammary gland tumors and human breast cancer. To determine whether this protein can be used as a diagnostic marker to detect the malignancy of mammary tumors, we also compared the pattern of SMP30 expression in mammary gland tumors with that in the normal mammary gland.

## 2. Results

### 2.1. Elevated SMP30 Expression Levels in Mammary Tumors Correspond to Their Malignancy

**Figure 1:**
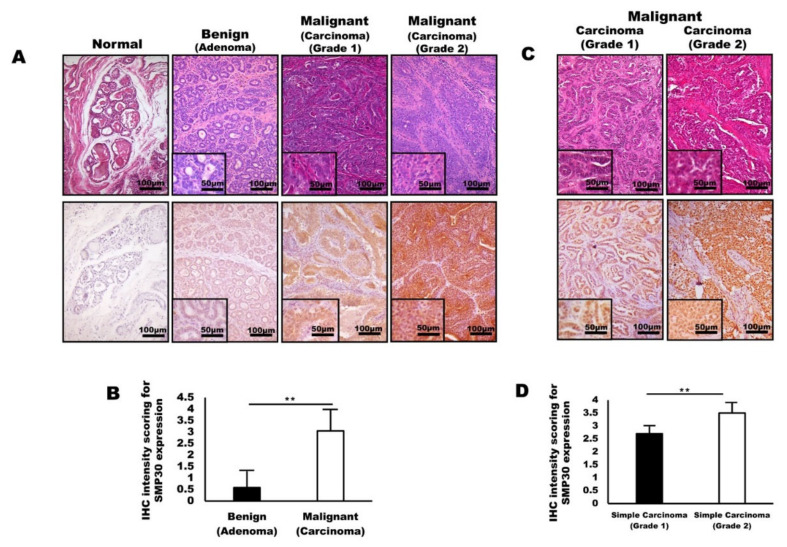
Senescence marker protein 30 (SMP30) levels in glandular epithelial cells correlate with malignancy of mammary gland tumors in dogs and cats. (**A**) Representative images of hematoxylin and eosin (H&E) staining and immunohistochemistry stained with anti-SMP30 normal tissue, adenoma, carcinoma (grade 1), and mammary carcinoma (grade 2). Significantly higher SMP30 expression was observed with an increase in malignancy of mammary gland tumors. Scale bars = 100 μm. Inset, scale bars = 50 μm. (**B**) Immunohistochemistry intensity scoring for SMP30 expression examined in canine mammary gland tumors. SMP30 expression significantly increased in carcinoma whereas adenoma showed a low level of SMP30 (** *p* < 0.01). (**C**) Representative images of H&E staining and immunohistochemistry of mammary specimens from felines with mammary carcinoma (grade 1) and mammary carcinoma (grade 2). A higher SMP30 expression level was observed in glandular epithelial cells of mammary carcinoma (grade 2) than that of mammary carcinoma (grade 1). Scale bars = 100 μm. Inset, scale bars = 50 μm. (**D**) Immunohistochemistry intensity scoring for SMP30 expression in feline mammary gland tumors. SMP30 expression significantly increased in grade 2 carcinoma compared to grade 1 carcinoma, indicating that SMP30 expression corresponds to the malignancy of neoplastic epithelial cells (** *p* < 0.01).

In immunohistochemistry, the tissue specimens collected from dogs with mammary tumors demonstrated that SMP30 protein levels were elevated dramatically with an increase in malignancy (Figure 1A). Weak patchy SMP30 positivity was restricted to the cytoplasm of neoplastic cells in mammary adenoma, whereas strong diffuse SMP30 positivity was observed in both the cytoplasm and nucleus of neoplastic cells in mammary carcinoma (Figure 1A). Interestingly, the mammary carcinoma tissue showed a higher expression of SMP30 (Figure 1B). However, mammary adenoma samples were characterized by significantly lower SMP30 expression (Figure 1B) (*p* < 0.01). Strong SMP30 expression was mainly observed where the neoplastic cells have prominent nucleoli, severe pleomorphism, high cellularity, and numerous mitotic figures. Based on the results, SMP30 expression levels in neoplastic epithelial cells seem to increase with the malignancy of tumor cells. We next examined whether SMP30 expression levels can indicate malignancy of feline mammary carcinoma. Immunohistochemistry staining for feline mammary carcinoma samples and histologic analysis were employed. Mammary glands from felines diagnosed with mammary carcinoma showed a significant increase in SMP30 expression levels in grade 2 mammary carcinoma compared with grade 1 mammary carcinoma (Figure 1C,D) (*p* < 0.01). Moreover, stronger nuclear and cytoplasmic expression levels of SMP30 were observed in proliferating neoplastic epithelial cells as the malignancy of mammary neoplasia increased.

### 2.2. SMP30 Expression Is Highly Associated with the Differentiation of the Tumor Cells in Various Types of Mammary Gland Tumors

**Figure 2:**
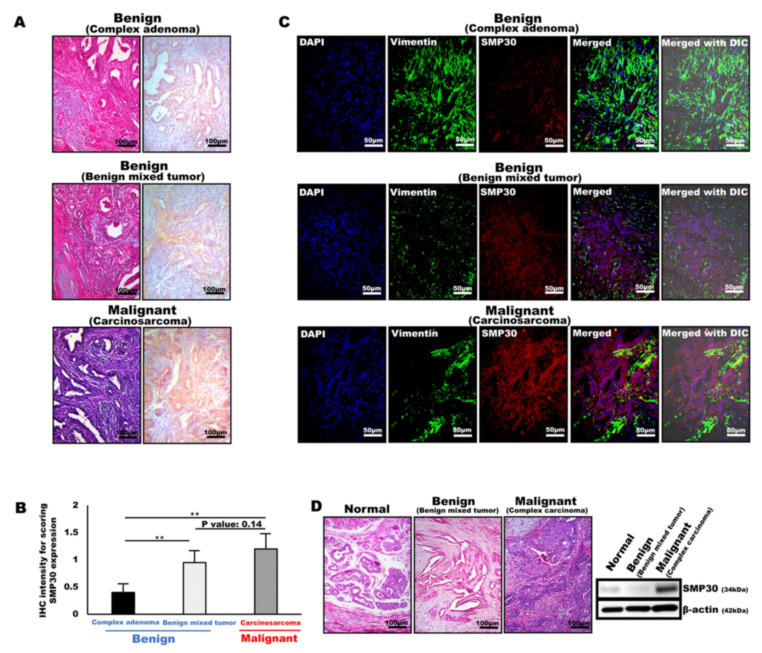
SMP30 expression correlates with the malignancy of glandular epithelial cells in various types of mammary gland tumors. (**A**) Representative H&E staining and immunohistochemistry images of SMP30 protein in benign mammary gland tumors (complex adenoma, benign mixed tumor) and malignant mammary gland tumor (carcinosarcoma). Scale bars = 100 μm. (**B**) Immunohistochemistry reactivity for SMP30 expression examined in various types of mammary gland tumors in dogs. SMP30 expression level was lowest in complex adenoma, but SMP30 expression level was increased by approximately threefold in carcinosarcoma, which shows the highest SMP30 expression level (** *p* < 0.01). Benign mixed tumor showed an intermediate SMP30 expression level, but SMP30 expression level was increased by nearly twofold compared with that of complex adenoma (** *p* < 0.01). (**C**) Representative immunofluorescence images of vimentin (green) and SMP30 (red). The nuclei were labeled by DAPI (blue). Merged image was shown with differential interference contrast (DIC). Any colocalization (yellow) of vimentin and SMP30 was not observed. Glandular epithelial cells with high levels of SMP30 expression appeared in carcinosarcoma, whereas low SMP30 expression levels appeared in complex adenoma and benign mixed tumor. Scale bars = 50 μm. (**D**) Representative H&E staining images and western blot analysis for SMP30 protein levels in normal mammary gland, benign mammary gland tumor (benign mixed tumor), and malignant mammary gland tumor (carcinosarcoma). β-actin was used as a loading control. SMP30 protein levels were low in normal and benign mixed tumor, whereas they were higher in carcinosarcoma.

SMP30 was specifically expressed in neoplastic epithelial cells, especially in the cells with malignant rather than benign neoplastic epithelial cells. Immunohistochemistry and immunofluorescence were performed to confirm the origin cells of SMP30 expressions using different types of tumor tissues composed of complex origins of both mesenchymal and epithelial origin cells, such as benign mixed tumor, carcinosarcoma, and complex adenoma. Interestingly, SMP30 was not expressed in any of the myoepithelial cell origin components, whereas SMP30 was specifically expressed in proliferative neoplastic glandular epithelial cells (Figure 2A). Microscopically, complex adenoma was mainly composed of proliferative myoepithelial cells and well-differentiated glandular epithelial cells (Figure 2A). Immunohistochemically, the well-differentiated neoplastic glandular epithelial cells were very weakly positive for SMP30 (Figure 2A,B). The normal architecture of the mammary gland in the benign mixed tumor was diffusely replaced by neoplastic glandular epithelial cells accompanied by the proliferation of myoepithelial cells forming a mature bone. Although some of the neoplastic cells were pleomorphic and showed high cellularity with prominent nucleoli, most of the neoplastic glandular epithelial cells were well differentiated (Figure 2A). In immunohistochemical staining, SMP30 was expressed diffusely, distributed only in neoplastic glandular epithelial cells, but the intensity was not strong compared with that of the carcinosarcoma (Figure 2A,B). However, the positivity was higher than that of adenoma, consistent with higher cellularity, more prominent nucleoli, and more severe cellular pleomorphism than the cells of adenoma (Figure 2A,B). The SMP30 expression levels in carcinosarcoma were the strongest among the other types of tumors (Figure 2A,B). Although the major components of the tumor were proliferative myoepithelial cells, remnant neoplastic glandular epithelial cells showed prominent nucleoli, severe pleomorphism, and high cellularity accompanied by frequent mitotic figures, indicating poor differentiation levels (Figure 2A). SMP30 expression significantly increased in both carcinosarcoma and benign mixed tumor compared with complex adenoma (threefold for carcinosarcoma (*p* < 0.01), twofold for benign mixed tumor (*p* < 0.01)) (Figure 2B). Double staining was performed using two different antibodies, anti-SMP30 (red) and anti-vimentin (green), for myoepithelial cell markers to visualize the localization of SMP30 expression in tumor tissues (Figure 2C). SMP30 was not merged with vimentin, indicating that SMP30 was not expressed in any of the myoepithelial cells in complex adenoma, benign mixed tumor, and carcinosarcoma. Moreover, SMP30 expression increased with the malignancy of mammary neoplasia; complex adenoma was the lowest, benign mixed tumor was the second, and carcinosarcoma was the highest, which was the same pattern as the immunohistochemical results (Figure 2C). Western blot analysis was performed to compare SMP30 protein levels among normal, benign, and malignant tumors. For the benign tumor, benign mixed tumor was chosen. In contrast, complex carcinoma sample was used for malignant mammary gland tumor. Microscopically, the pleomorphism of the glandular epithelial cells was minimal in benign mixed tumor, whereas the glandular epithelial cells in complex carcinoma showed prominent nucleoli and severe pleomorphism (Figure 2D). In accordance with the histopathological features, SMP30 protein was weakly detected in normal mammary glands. Moreover, the SMP30 protein level of complex carcinoma was the highest in western blot analysis. However, the SMP30 protein level in benign mixed tumor was significantly lower than that of complex carcinoma, indicating that SMP30 protein was rarely expressed in well-differentiated glandular epithelial cells (Figure 2D). In accordance with protein levels, SMP30 mRNA levels were significantly higher in carcinoma than in normal mammary gland (Figure S2, Supplementary Materials).

### 2.3. SMP30 Expressions of Canine Primary Carcinoma Cells Are Colocalized with Pan-Cytokeratin, a Marker of Epithelial Origin in Mammary Glands

**Figure 3:**
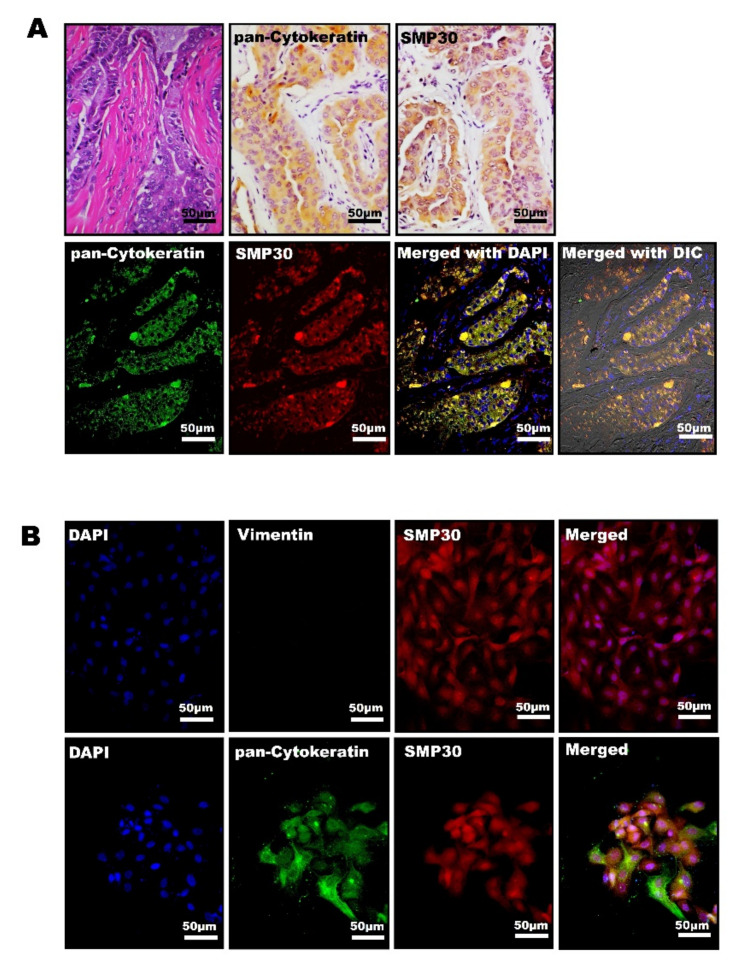
Neoplastic glandular epithelial cells with high levels of SMP30 correlate positively with pan-cytokeratin in canine primary mammary carcinoma cells. (**A**) Representative images of H&E staining and immunohistochemistry for SMP30 and pan-cytokeratin in a canine mammary simple carcinoma tissue used for primary cell isolation. SMP30 and pan-cytokeratin were expressed in neoplastic epithelial cells. Scale bars = 50 μm (top). Representative immunofluorescence images of pan-cytokeratin and SMP30 in simple carcinoma tissues. In the neoplastic glandular epithelial cells, colocalization of pan-cytokeratin and SMP30 was observed. Scale bars = 50 μm (bottom). (**B**) Representative immunofluorescence images of vimentin (green) and SMP30 (red) in primary cells of canine mammary carcinoma. DAPI (blue) was used for nuclei labeling; vimentin was negative whereas SMP30 was expressed strongly both in the cytoplasm and nucleus of the cells. Scale bars = 50 μm (top). Representative immunofluorescence images of pan-cytokeratin (green) and SMP30 (red) in canine mammary carcinoma primary cells. The nuclei were stained with DAPI (blue). Colocalization (yellow) of pan-cytokeratin with SMP30 was observed, and both were strongly expressed in the cytoplasm and nucleus of the cells. Scale bars = 50 μm (bottom).

The localization pattern of SMP30 in mammary carcinoma was next confirmed using primary isolated mammary carcinoma cells in vitro and histopathological analysis. Adenocarcinoma cells were isolated from a dog diagnosed as having a simple carcinoma. In immunohistochemistry, adenocarcinoma cells showed a strong positive reaction for pan-cytokeratin and those pan-cytokeratin-positive cells were also strongly positive for SMP30 (Figure 3A). Immunofluorescent staining showed marked colocalization of SMP30 (red) with pan-cytokeratin (green). Both pan-cytokeratin and SMP30 levels were highly expressed in neoplastic glandular epithelial cells (Figure 3A). In immunofluorescence using primary isolated mammary cancer cells incubated with SMP30, pan-cytokeratin, and vimentin antibodies, primary carcinoma cells were negative for vimentin (green) (Figure 3B). In contrast, high levels of SMP30 (red) were observed in both the cytoplasm and nucleus in the neoplastic epithelial cells (Figure 3B). In immunofluorescence using pan-cytokeratin (green) and SMP30 (red) labeling on these primary cells, only yellow colors (red merged with green areas) were considered colocalization with a high SMP30 expression level and pan-cytokeratin. Interestingly, both pan-cytokeratin and SMP30 were exactly overlapped, indicating that SMP30 proteins were mainly expressed in epithelial cells in the tumor, such as mammary glandular neoplastic cells (Figure 3B).

### 2.4. SMP30 Levels Increased with Malignancy in Human Breast Cancer Cells

**Figure 4:**
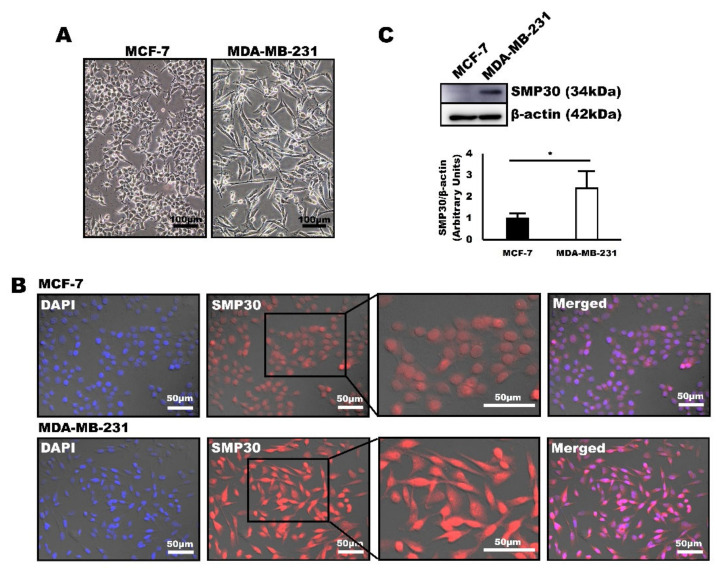
SMP30 protein expression is significantly stronger in a poorly differentiated human breast cancer cell line, MDA-MB-231. (**A**) Representative images of human breast cancer cells, MCF-7 and MDA-MB-231. Scale bars = 100 μm. (**B**) Representative immunofluorescence images of SMP30 expressed in MCF-7 and MDA-MB-231. MDA-MB-231, which is a poorly differentiated mammary carcinoma cell line, was characterized by a significantly stronger SMP30 protein expression than that of MCF-7, which is a well-differentiated mammary carcinoma cell line. Scale bars = 50 μm. (**C**) Protein levels of SMP30 and β-actin were assessed by western blot analysis in MCF-7 and MDA-MB-231. SMP30 expression was significantly higher in MDA-MB-231. (* *p* < 0.05)

Human carcinoma cell lines MCF-7 and MDA-MB-231 were used for western blot and immunofluorescence assays to determine whether SMP30 can be used as a diagnostic marker in human breast cancer. MCF-7 is known for being a well-differentiated mammary carcinoma cell line, whereas MDA-MB-231 is known for its mostly poor differentiation characteristics [33]. Our cultured MDA-MB-231 cells also showed severe pleomorphism, high cellularity, and prominent nucleoli, whereas MCF-7 cells were characterized by low pleomorphism, moderate cellularity, and inconspicuous nucleoli (Figure 4A). Immunofluorescence was first performed to detect the SMP30 expression from each cell to examine whether SMP30 expression correlates with malignancy of mammary carcinoma in human cell lines. Interestingly, higher SMP30 levels were detected in MDA-MB-231 cells with a higher malignancy than in MCF-7 cells with a lower malignancy (Figure 4B). Western blot analysis was performed to quantify the SMP30 expression level between MCF-7 and MDA-MB-231. Consistent with immunofluorescence, SMP30 expression level was higher in MDA-MB-231 than that of MCF-7, indicating that SMP30 expression increased with malignancy in human cells (Figure 4C).

### 2.5. P-ERK Expression Corresponds to SMP30 Expression in Canine Mammary Gland Tumors and Human Breast Cancer Cells

**Figure 5:**
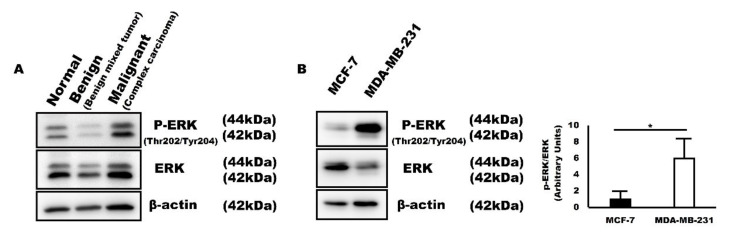
p-ERK protein expression is consistent with SMP30 in canine mammary gland tumors and human breast cancer cell lines, MCF-7 and MDA-MB-231. (**A**) p-ERK expression levels were low in normal mammary gland and benign mammary gland tumor, whereas they were high in malignant tumor. (**B**) p-ERK expression levels increase with malignancy in cancer cells. (* *p* < 0.05).

ERK is known to increase in cancer cells, especially in chemotherapy-resistant cancer cells. It is a key mediator of antiapoptosis and promotes cell proliferation and cell survival [30]. Moreover, it has been reported that SMP30 is upregulated by the ERK signaling pathway in damaged organs [28,29]. For this reason, we next evaluated ERK expression levels to double confirm that increased SMP30 levels are related to proliferation and antiapoptosis of cancer cells. Interestingly, in western blot analysis, p-ERK, an active form of ERK, expression levels were consistent with SMP30 expression (Figure 5). In canine mammary gland tumors, p-ERK expression increased with the malignancy of glandular epithelial cells. p-ERK expression was low in normal mammary glands and benign mixed tumor. However, p-ERK expression was the highest in complex carcinoma composed of poorly differentiated neoplastic glandular epithelial cells (Figure 5A). The pattern was also reproduced in human breast cancer cell lines. p-ERK expression level was higher in MDA-MB-231 than in MCF-7, which was the exact same pattern observed in SMP30 expression (Figure 5B).

### 2.6. SMP30 Expression Levels in Human Breast Cancer Tissues and Its Correlation with Age and Histopathological Grades of Carcinomas

**Figure 6:**
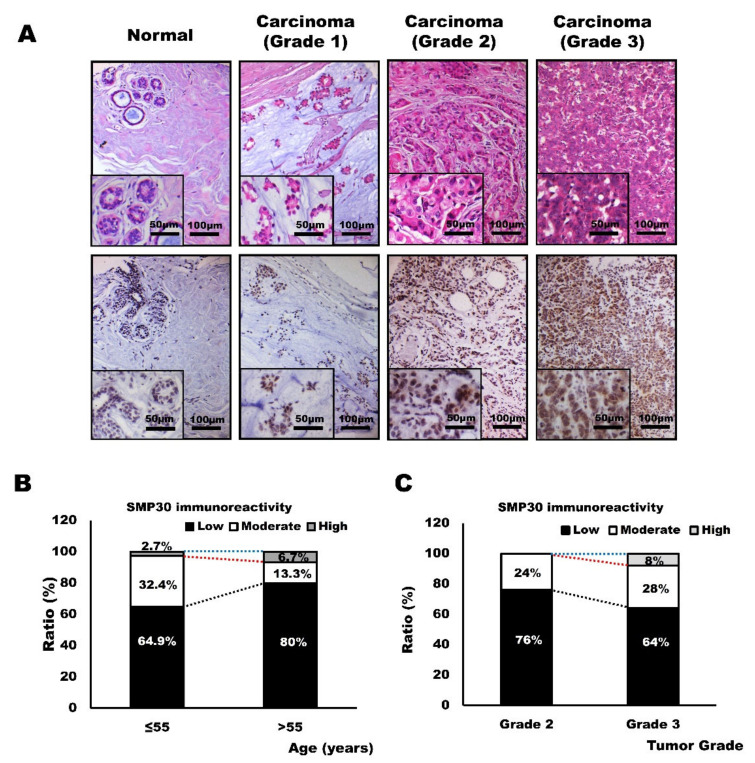
SMP30 protein expression in human mammary carcinoma and its relationship with age and grade of tumors. (**A**) Representative images of H&E staining and immunohistochemistry of human breast tissues. SMP30 expression was undetectably low in normal mammary glands. Weak to moderate immunoreactivity was noted in grade 1 carcinoma. Increased expression of SMP30 was noted in grade 2 carcinoma and strong and intense immunoreactivity of SMP30 expression was observed in grade 3 carcinoma. Scale bars = 100 μm. Inset, scale bars = 50 μm. (**B**) SMP30 expression decreases with age. (**C**) SMP30 expression increases with histopathological grades.

We next observed the SMP30 expression in human breast cancer tissues to confirm the utility of SMP30 as a diagnostic marker. SMP30 expression was undetectably low in normal breast tissue, as shown in animal mammary gland tissue specimens. However, in grade 1 carcinoma, low to moderate expression of SMP30 was observed in neoplastic glandular epithelial cells. In samples with grade 2 carcinoma, moderate SMP30 expression was observed. Strong and intense expressions of SMP30 were observed only in grade 3 mammary carcinoma with high cellularity and prominent nucleoli with severe pleomorphism, which was the same pattern shown in canine and feline mammary gland tumors (Figure 6A). Because malignant cancer usually occurs after the age of 55 years and as many cohort studies do [34,35], the age group was divided into a young patient group with an age of ≤55 years and an old patient group with an age of >55 years. Most SMP30 expressions were low (80%, 12 of 15 cases) in the old patient group, whereas 64.9% (24 of 37) of SMP30 immunoreactivity was observed in the young patient group (Figure 6B). Moderate expression was also higher in the younger patient group, with 32.4% (12 of 37) of reactivity, whereas only 13.3% (two of 15) of SMP30 expression was observed in the old patient group (Figure 6B). High expression of SMP30 was noted in only one case in each group (2.7% (one of 37) vs. 6.7% of the old patient group (one of 15)); hence, it was difficult to compare the expression pattern between the groups. Then, we evaluated SMP30 immunoreactivity based on tumor grades (Figure 6C). As tumor grades increase, SMP30 expression tends to increase. Grades 1 and 2 carcinomas only showed low to moderate immunoreactivity with SMP30. High immunoreactivity of SMP30 was observed only in grade 3 carcinomas, with 8% of immunoreactivity (two of 25) (Figure 6C). Although grade 1 carcinomas showed 50% low immunoreactivity (one of two) and 50% moderate immunoreactivity (one of two), the number of samples was too small, and it was difficult to confirm the expression pattern. However, when grade 2 carcinomas were compared with grade 3 carcinomas, grade 2 carcinomas showed higher rates of low expression of SMP30 (76% (19 of 25) vs. 64% (16 of 25)) and lower rates of moderate expression of SMP30 (24% (six of 25) vs. 28% (seven of 25)) (Figure 6C). These results indicate that SMP30 expression also increases with the malignancy of breast cancer in humans. Clinical information of the patients, diagnosis, and grade of carcinoma samples used for analysis in this study are presented in Table S1, Supplementary Materials.

## 3. Discussion

The most common risk factors of animal mammary gland tumors are age predisposition, ranging from 8 to 10 years, small breed, and spay status [7,36,37]. In our study, a total of 42 dogs with mammary gland adenoma and carcinoma were traced in this study. The details of the diagnosis of tumors and signalments, including species, breed, and sex of the cases, are presented in Table S2. Similarly, in our retrospective study based on clinical information of 42 dogs, both benign and malignant mammary gland tumors are usually developed in small pure breeds (Figure S1 and Table S2, Supplementary Materials). All adenomas tended to occur in small breeds, such as Maltese (n = 3, 21.4%), Shih Tzu (n = 3, 21.4%), Dachshund (n = 2, 14.3%), Yorkshire Terrier (n = 2, 14.3%), Poodle (n = 2, 14.3%), and Miniature Pinscher (n = 2, 14.3%) with an age of >8 years (Figure S1). Although some larger breeds of dogs, such as Siberian Husky and Jindo dogs, were reported, carcinoma tended to also occur mostly in small breeds. The predominance was found in Malteses (n = 11, 39.3%) compared with other small breeds, such as Cocker Spaniel (n = 4, 14.3%), Poodle (n = 2, 7.1%), and Shih Tzu (n = 2, 7.1%) (Figure S1). However, other risk factors such as age and especially spay status tend to be more related to the occurrence of malignant tumors than benign tumors (Figure S1, Supplementary Materials). These retrospective results indicate that mammary gland tumors found in elderly or intact bitches are more likely to be malignant. This could be related to clinical problems because the incidence of mammary gland tumors has increased owing to the longer life span based on humanized lifestyle and better medical treatment of domestic animals [36,38]. This problem can be more severe in countries like Europe where ovariohysterectomy is not frequently performed, because the incidence of mammary gland tumors is reported to be 50% to 70% of all neoplasias developed in intact bitches [6,7,39].

Clinical presentation of mammary gland tumors is usually not well recognized, and many animals already have more than one tumor at the time of clinical signs [7]. Therefore, early diagnosis is the most effective treatment for mammary gland tumors. This also applies to human breast cancer. Histopathological analysis is the most definitive way to diagnose mammary gland tumors. However, it is not easy to differentiate between benign and malignant tumors if the malignancy is at an early stage; hence, it is helpful to use biomarkers for diagnosis. The most well-known and widely used markers are Ki-67, endothelial growth factor receptor, ERs, PRs, and HER2 [10,11,13,36]. Human markers for the diagnosis of breast cancer can also be used in cases of domestic animals because the aspects of mammary gland tumors found in domestic animals are analogous to those of human breast cancer [36,40]. In particular, ERs, PRs, and HER2 are highly recommended biomarkers in both humans and domestic animals [13,14,36]. However, there are still some obstacles in the use of these markers as diagnostic markers that can diagnose mammary gland tumors in various species. HER2 expression varies from 7.6% to 31.6% of human breast cancer [41], and the intensity in animals also varies [16,42]. ERs and PRs show a very high positivity and are strong predictors of prognosis of endocrine therapy in human breast cancer [13,14,41]. However, they are unreliable markers in domestic animals because of a lack of knowledge for the prognosis of endocrine therapy and the huge variability of their results [15,16]. For example, the ER is known to be highly affected by age and spay and pseudopregnancy status [7]. Therefore, it is still important to find diagnostic markers that are reliable predictors to help in therapeutic decision making but inexpensive and easily accessible in domestic animals.

Recently, it has been suggested that SMP30 is pivotal for cell survival and cytoprotection [23,24,25,26], by preventing oxidative stress, and apoptosis of cells in many organs [23]. One of the main mechanisms of SMP30 in this process is scavenging of ROS by the generation of vitamin C [23,26]. High doses of ROS activate the cell death process and oxidative DNA damage [43,44]; thus, it is important to inhibit ROS for cell survival. Several studies revealed that SMP30 deficiency results in high levels of ROS generation and exacerbates oxidative stress and apoptosis in many parenchymal organs such as the liver, heart, brain, and lungs [22,23,26,45,46]. These studies suggest that SMP30 and its product, vitamin C, are pivotal for cell survival. Ironically, ROS is also the key defense mechanism against cancer cells by inflicting cellular damage to carcinogenic cells [47,48]. Cancer cells detoxify ROS by expressing antioxidant proteins for their survival; thus, the death signaling pathway is impaired in advanced stages of tumors [49,50]. Furthermore, many studies suggest the possibility of using ROS as an anticancer therapy [50,51,52]. These results suggest that SMP30 and vitamin C are also important survival factors for tumorigenic cells; thus, they are not helpful in eliminating or alleviating cancer after its development. Interestingly, in our study, MCF-7 cells with lower SMP30 expression levels than MDA-MB-231 cells show significantly higher expression level of Bax, an apoptotic marker, than in MDA-MB-231 cells, whereas the expression level of PCNA, a cell proliferation marker, was significantly lower than in MDA-MB-231 cells. Moreover, knockdown of SMP30 in MCF-7 cells dramatically increased cell death under ROS damage which is consistent with the hypothesis (Figure S3, Supplementary Materials).

Although the role of SMP30 and its concomitant expression are well documented in other tumors, such as hepatocellular carcinoma [53], the relationship between SMP30 and mammary gland tumors still remains ambiguous and controversial. Sar et al. reported that high SMP30 expression is noted in MCF-7 cells [31]. However, Maia et al. reported that SMP30 expression was highest in normal glandular epithelial cells and decreased with the malignancy of mammary gland tumors [32]. In our study, SMP30 expression strikingly increases in malignant mammary tumors. SMP30 expression levels were significantly lower in the normal and benign mammary gland tumor tissues. Moreover, SMP30 mRNA expression was also significantly higher in mammary carcinoma than in normal mammary glands (Figure S2, Supplementary Materials). Consequently, we found that SMP30 expression is more related to the proliferation than differentiation or invasiveness of neoplastic cells. Prominent nucleoli and high mitotic index are the indicators of proliferation, and SMP30 is generally expressed much more strongly in neoplastic cells with these indicators. This pattern is usually observed in malignant mammary tumors because there are many more proliferative neoplastic glandular cells in these tumors than in benign tumors. However, SMP30 expression is low or unobserved in malignant mammary gland tumors with severe necrosis accompanied by apoptosis of neoplastic glandular epithelial cells (data not shown).

In humans, young patients tend to develop breast cancer less than older patients [54]. However, when it happens, young patients have a worse prognosis than older patients [54]. They have much lower 5-year survival rates because of the more aggressive and advanced characteristics of tumors [55]. This outcome may be linked with SMP30 expression. As its name suggests, SMP30 expression level dramatically decreases with senescence. Therefore, SMP30 expression is markedly high at a young age [56]. SMP30 can prevent cancer development by generating the strong antioxidant vitamin C. However, after cancer development, cells in young tissues proliferate and regenerate more easily than in older tissues because SMP30 also has an important role as a cell survival factor. Consistently with this hypothesis, many studies revealed that tumor grade is much higher with higher proliferation and nodal invasiveness of tumor cells in younger patients than in older patients [57,58]. Thus, we assumed that excessive SMP30 expression is associated with diminished apoptosis, uncontrolled proliferation, and immortality of tumor cells. Therefore, strong SMP30 expression is generally noted in malignant mammary gland tumors with prominent nucleoli and many mitotic figures. This was also observed in our study, although the pattern was weaker than in animal mammary gland tumors because of insufficiency of grade 1 carcinoma samples (Figure 6C). The pattern was also reproduced in human mammary carcinoma cell lines MCF-7 and MDA-MB-231. SMP30 showed relatively weak expression in MCF-7, which is a well-differentiated mammary carcinoma cell line. However, MDA-MB-231, a poorly differentiated mammary carcinoma cell line, showed intense SMP30 expression both in western blot analysis and immunofluorescence assays (Figure 4). To confirm our hypothesis, we observed the expression patterns of p-ERK, which is a survival factor mediating antiapoptosis and cell proliferation [30]. p-ERK expression has been reported to be associated with the upregulation of SMP30 in oxidatively damaged brains [28,29], but the link between SMP30 and p-ERK in mammary gland tumors has not been well defined. Interestingly, expression patterns of p-ERK were to a large extent in accordance with those of SMP30 (Figure 5), which strengthens our hypothesis. 

In summary, the current study is the first to establish a correlation between SMP30 expression and mammary gland tumors originating from glandular epithelial cells. In our study, SMP30 was sensitive and highly specific for detecting proliferative glandular epithelial cells with malignancy. Moreover, the expression pattern of SMP30 in MCF-7, MDA-MB-231, and human breast cancer tissues suggested a possibility that SMP30 could be a useful diagnostic marker and therapeutic target for breast cancer in humans. However, the precise underlying mechanism and the expression pattern of SMP30 in more human mammary gland tissues, especially with a low grade of carcinoma or benign mammary gland tumor, need to be investigated more in further studies.

## 4. Materials and Methods

### 4.1. Tissue Samples and Histopathologic Examination

A total of 42 canine and 2 feline mammary gland tumor tissue samples were obtained through total mastectomy by local animal hospitals and the Department of Veterinary Surgery (College of Veterinary Medicine, Kyungpook National University) for diagnosis and were diagnosed by the Department of Veterinary Pathology (College of Veterinary Medicine, Kyungpook National University). As controls, 2 normal mammary gland tissues were collected from each dog. In addition, records of each animal, such as breed, sex, age, clinical information, and neutralization, were provided (Figure S1 and Table S2, Supplementary Materials). All excised mammary gland tumor tissues were 10% neutral formalin fixed, paraffin embedded (FFPE), sectioned to 4 µm thickness, and stained with hematoxylin and eosin (H&E) for microscopic examination. Tumor grading was based on the classification suggested by Goldschmidt et al. [7]. The classification is composed of 3 criteria which are tubule formation, nuclear pleomorphism, and mitoses per 10 high power field. Firstly, for tubule formation, the formation of tubules in >75% is 1 point, tubule formation in 10–75% is 2 points, and tubule formation in <10% is 3 points. For nuclear pleomorphism, inconspicuous small nucleus and occasional nucleoli is 1 point, moderate degree of variation in nuclear size and shape, hyperchromatic nucleus, and presence of nucleoli is 2 points, and marked variation in the size of the nucleus, hyperchromatic nucleus, and one or more prominent nucleoli is 3 points. For mitoses per 10 high power field, 0–9 mitoses/10 high power field is 1 point, 10–19 mitoses/10 high power field is 2 points, and ≥20 mitoses/10 high power field is 3 points. Histologic malignancy grade is based on the total scores of these 3 criteria. A total score of 3–5 is grade 1 tumor, 6–7 is grade 2 tumor, and 8–9 is grade 3 tumor. However, we also considered invasiveness into blood vessels and lymphatic ducts for the diagnosis, considering clinical importance. A total of 19 canine mammary gland tumors were selected as the most representative samples, and immunohistochemistry staining was performed on them for further analysis. Human breast tissue microarrays (TMAs) were purchased from a commercial supplier (US Biomax, Inc., Rockville, MD, USA). The clinical information for carcinoma samples used for immunohistochemistry analysis is summarized in Table S2. In the case of human tissue, we had received all clinical information of patients, including tumor grades, from US Biomax where we bought TMA slides. All the experiments and protocols using animal tissues used in this study were approved by the Kyungpook National University Institutional Animal Care and Use Committee (IACUC, approval number 2020-0139). Human samples of breast cancer were purchased from US Biomax, Inc., and US Biomax, Inc. follows standard medical care and protects the privacy of the donors. All human tissue samples were collected under HIPAA-approved protocols.

### 4.2. Immunohistochemistry and Immunoreactivity Evaluation

The Histostain^®^ Plus Broad Spectrum Kit (Cat#859043, Life Technologies, Carlsbad, CA, USA) was used for immunohistochemistry. For antigen retrieval, deparaffinized serial tissue sections were treated with 3% hydrogen peroxide diluted in methyl alcohol at room temperature for 35 min and heated in citrate buffer (pH of 6.0) for 30 min. To prevent nonspecific reactions, the sections were treated with a blocking solution (Cat#859043, Life Technologies, Carlsbad, CA, USA) at room temperature for 1 h. Then, the sections were incubated at 4 °C overnight with primary antibody rabbit anti-SMP30 (1:200, Cat#SML-ROI001-EX, Cosmo Bio Co., Ltd., Tokyo, Japan), and mouse anti-pan-cytokeratin (1:200, Cat#ab86734, Abcam, Cambridge, UK). The sections were washed 3 times using phosphate-buffered saline (PBS) and reacted with broad-spectrum secondary antibody (Cat#859043, Life Technologies, Carlsbad, CA, USA) and with horseradish peroxidase (HRP)-conjugated streptavidin (Cat#859043, Life Technologies, Carlsbad, CA, USA) for 10 min. Finally, the reactions were visualized using a 3,3-diaminobenzidine (DAB) peroxidase substrate kit (Cat#SK-4100, Vector Laboratories, Inc., Burlingame, CA, USA), followed by counterstaining with hematoxylin for 30 s.

In the case of a human TMA slide, before deparaffinization, the slide was baked at 60 °C for 30 min following the manufacturer’s guidelines. After the deparaffinization, the slide was incubated with proteinase K diluted in 1× Tris-EDTA buffer for 20 min at 37 °C. The slide was washed 3 times using PBS. Then, immunohistochemistry was performed under the same protocol used in other tissue slides. The SMP30 dilution for the TMA slide was diluted in PBS at 1:50.

SMP30 levels in immunohistochemistry were scored based on Table 1 shown below. The immunoreactive scores (IRSs) of SMP30-positive stained cells were evaluated over 5 fields at a power of ×200. To generate the total immunoreactive scores (IRSs), both the staining intensity and positive cell ratio were considered. For the staining intensity, no staining was considered 0, 1 for weak, 2 for moderate, 3 for strong, 4 for intense. The positive cell ratio scoring criteria included 8 categories: 0 for <5% positive cells, 0.5 for 5% to 15% positive cells, 1 for 16% to 25% positive cells, 1.5 for 26% to 33% positive cells, 2 for 34% to 50% positive cells, 2.5 for 51% to 66% positive cells, 3 for 67% to 75% positive cells, 4 for 76% to 100% positive cells. The average of these two scores provided the final IRS. These immunostaining results were scored by two pathologists independently. The same scoring criteria were applied to all obtained canine and feline mammary tumor samples.

The SMP30 expression categories for human TMA samples were low (1+), moderate (2+), and high (3+). The results were double confirmed by using Image J/Fiji software version 1.53c (National Institutes of Health, Bethesda, MD, USA).

### 4.3. Primary Cell Isolation of Canine Mammary Adenocarcinoma

The tissue collected by the Department of Veterinary Surgery was rinsed 5 times with Dulbecco’s phosphate-buffered saline (DPBS; Gibco, Rockville, MD, USA) containing 1% penicillin–streptomycin (10,000 IU and 10,000 μg/mL, respectively; Gibco, Rockville, MD, USA) and evenly cut. One tissue was immediately fixed in 3.7% paraformaldehyde (PFA; Sigma-Aldrich, St. Louis, MO, USA) and histopathological analysis was performed by the Department of Veterinary Pathology (College of Veterinary Medicine, Kyungpook National University), and another was employed for primary cell isolation. For the cell isolation, the tissue was mechanically minced into 1–3 mm^2^, digested in DPBS containing 0.1% collagenase type IV (Sigma-Aldrich, St. Louis, MO, USA) at 37 °C for 40 min, and filtered through a 40 μm cell strainer (BD Falcon, Franklin Lakes, NJ, USA) to obtain single cell populations. After that, single cells were cultured in advanced Dulbecco’s modified Eagle’s medium (DMEM; Gibco, Rockville, MD, USA) containing 10% fetal bovine serum (FBS; Gibco), 1% L-glutamine (GlutamaxTM; Gibco, Rockville, MD, USA), and 1% penicillin–streptomycin (Gibco, Rockville, MD, USA) at 37 °C in a humidified atmosphere with 5% CO_2_. Once the adherent cells on the culture dishes were identified, the supernatant was removed, and exchanged for fresh culture media. The cells were expanded until passage 5 for further analysis.

### 4.4. Cell Culture

Human breast adenocarcinoma cell lines MCF-7 and MDA-MB-231 were cultured in DMEM (WELGENE Inc., Gyeongsan, Korea) supplemented with 10% heat-inactivated FBS (Invitrogen, Carlsbad, CA, USA) and penicillin–streptomycin–amphotericin B solution (WELGENE Inc., Gyeongsan, Korea). The cells were plated at 5 × 10^5^ cells in 6-well plates and were incubated at 37 °C overnight. Whole cells were harvested using 1× electrophoresis sample buffers diluted in the lysis buffer supplemented with 0.1 mM sodium vanadate, protease inhibitors (Roche Diagnostics, Mannheim, Germany), Pefabloc SC (Roche Diagnostics, Mannheim, Germany), sodium fluoride, and sodium pyrophosphate.

### 4.5. Western Blot Analysis

The protein of snap-frozen mammary gland tissues was extracted with lysis buffer containing 0.1 mM Na3VO4, protease inhibitors (Roche Diagnostics, Mannheim, Germany), Pefabloc SC (Roche Diagnostics, Mannheim, Germany), sodium fluoride, and sodium pyrophosphate. A Bio-Rad protein assay (Bio-Rad Laboratories, Hercules, CA, USA) was used to measure protein concentrations. Extracted tissue and cell proteins were boiled at 100 °C for 10 min and were centrifuged at 6000 RPM. Equal amounts of samples were loaded and separated by sodium dodecyl sulfate–polyacrylamide gel electrophoresis (SDS-PAGE) and transferred to polyvinylidene difluoride (PVDF) membranes (MilliporeSigma, Billerica, MA, USA). After that, blocking was performed with 5% skim milk diluted in Tris-buffered saline containing 0.1% Tween-20 (TBS/T) for 1 h. The proteins were analyzed by immunoblotting with primary antibody rabbit anti-SMP30 (1:2000; Cosmo Bio Co., Ltd., Tokyo, Japan), anti-phospho-ERK (1:1000; Cat#9101, Cell signaling, Danvers, MA, USA), and anti-ERK1/2 (1:1000; Cat#sc-514302, Santa Cruz Biotechnology, Santa Cruz, CA, USA). After being washed by TBS/T, the membranes were incubated with HRP-conjugated goat anti-rabbit (Cat#401393, Calbiochem, San Diego, CA, USA) or goat anti-mouse (Cat#401253, Calbiochem, San Diego, CA, USA) for 1 h at room temperature. The samples were visually quantified using the ProNATM ECL Ottimo (Translab, Seoul, Republic of Korea,) and AmershamTM Imager 680 (GE Healthcare Björkgatan, Uppsala, Sweden). A loading control was confirmed by mouse anti-β-actin (1:2000; Cat#a1978, Sigma-Aldrich, St. Louis, MO, USA).

### 4.6. Immunofluorescence

We used rabbit anti-SMP30 (1:200; Cosmo Bio Co., Ltd., Tokyo, Japan) conjugated with 555 fluorochrome (colored by red) and mouse anti-pan-cytokeratin (1:200; Abcam, Cambridge, UK) or mouse anti-vimentin (1:200; Cat#MA5-11883, Thermo Fisher Scientific, Inc., Waltham, MA, USA) conjugated with 488 fluorochrome (colored by green) for the immunofluorescence of mammary tumor tissues or tumor cells including MCF-7, MDA-MB-231, and isolated primary canine mammary carcinoma cells. The same was done for immunohistochemistry, FFPE tissues sectioned of 4µm were deparaffinized, and antigen retrieval with 3% hydrogen peroxide diluted in methyl alcohol and citrate buffer (pH of 6.0) was performed. After being cooled down at room temperature for 2 h, the sections were treated with 0.1% Triton X-100 diluted in PBS for 10 min for permeabilization. In the case of cells, MCF-7, MDA-MB-231, and isolated primary canine mammary carcinoma cells were plated on coverslips in 12-well plates and fixed in 4% PFA for 20 min. After being washed with PBS 3 times, the cells were permeabilized with 0.1% Triton X-100 diluted in PBS for 10 min. After being blocked with 5% donkey serum for 1 h, the sections or cells were incubated with primary antibodies including rabbit anti-SMP30 (1:200; Cosmo Bio Co., Ltd. Tokyo, Japan), mouse anti-pan-cytokeratin (1:200; Abcam, Cambridge, UK), and mouse anti-vimentin (1:200; Thermo Fisher Scientific, Inc., Waltham, MA, USA) overnight at 4 °C. After being washed with PBS 3 times each for 5 min, the samples were treated with the second antibody including Alexa Fluor 488 donkey anti-mouse IgG (1:500; Cat#ab150105, Abcam, Cambridge, UK) and Alexa Fluor 555 donkey anti-rabbit IgG (1:500; Cat#ab150066, Abcam, Cambridge, UK) for 1 h at room temperature. ProLong^®^ Gold Antifade Reagent with DAPI (Cat#8961, Cell Signaling, Danvers, MA, USA) was used for nuclei labeling. To omit the background staining, negative control slides without the first antibodies were produced with both cell and tissue samples. An Olympus BX53 fluorescence microscope (Olympus, Tokyo, Japan) was used for visualization.

### 4.7. Statistical Analysis

All obtained data from the experiments were expressed as mean ± standard deviation and statistical significance among the groups was determined based on unpaired Student’s *t*-tests, Mann–Whitney U tests or nonparametric one-way analysis of variance (ANOVA/Kruskal–Wallis test). *p* < 0.05 was considered statistically significant.

## Figures and Tables

**Table 1:** Grading standard for SMP30 level in immunohistochemistry.

Immunohistochemical Grading Standard for SMP30 Expression			
SMP30 Intensity (per 200× Field)	Score	SMP30 Positive Cell Ratio (per 200× Field)	Score
Negative	0	<5%	0
Weak	1	5–15%	0.5
Moderate	2	16–25%	1
Strong	3	26–33%	1.5
Intense	4	34–50%	2
		51–66%	2.5
		67–75%	3
		76–100%	4

## Supplementary Materials

The following are available online at https://www.mdpi.com/1422-0067/22/5/2340/s1.

## Abbreviations

BID	Bis in die
DAB	3,3-diaminobenzidine
DMEM	Dulbecco’s modified Eagle’s medium
DPBS	Dulbecco’s phosphate-buffered saline
ER	Estrogen receptor
FBS	Fetal bovine serum
FFPE	Formalin fixed, paraffin embedded
H&E	Hematoxylin and eosin
HER2	Human epidermal growth factor 2
HRP	Horseradish peroxidase
LPS	Lipopolysaccharide
NO	Nitric oxide
PBS	Phosphate-buffered saline
PFA	Paraformaldehyde
PR	Progesterone receptor
PVDF	Polyvinylidene difluoride
ROS	Reactive oxygen species
SDS-PAGE	Sodium dodecyl sulfate–polyacrylamide gel electrophoresis
SMP30	Senescence marker protein 30
TBS	Tris-buffered saline
TMA	Tissue microarray
TNF-α	Tumor necrosis factor alpha
