# Supplementary material for: Senescence Marker Protein 30 (SMP30): A Novel Pan-Species Diagnostic Marker for the Histopathological Diagnosis of Breast Cancer in Humans and Animals
Source: Int J Mol Sci. 2021 Feb 26;22(5):2340. doi: 10.3390/ijms22052340 (PMC7956281; doi:10.3390/ijms22052340)
Supplement: Supplementary file 1 [file ijms-22-02340-s001.zip › Supplementary materials (210213).doc]

**Supplementary Figure S1.** Epidemiology and retrospective study for association with known risk factors of mammary gland tumors in dogs. (A) Breed susceptibility, sex, and age of adenoma and carcinoma that occurred in dogs. All the adenoma cases occurred in small breeds such as Maltese, Dachshund, Yorkshire Terrier, Poodle, Miniature Pinscher, and Shih Tzu, mostly with the age of >8 years. Ovariectomy did not contribute to the prevention of adenoma development. (B) Most carcinoma cases occur in small breeds with a predominance in Maltese. However, large breeds such as Jindo dog and Siberian Husky were also reported. Age was a riskier factor in carcinoma than that of adenoma. Ovariectomy was important for preventing carcinoma contrary to adenoma.

**Supplementary Figure S2.** Relative mRNA expression of SMP30 of canine normal mammary gland and canine mammary carcinoma. (A) The bar chart for SMP30 mRNA expression quantification between canine normal mammary gland and canine mammary carcinoma. SMP30 mRNA was higher in canine mammary carcinoma than that of normal mammary gland. (B) Primer sequence of SMP30 and internal standard HPRT gene for qPCR. The sequence of HPRT was referred from Johnson, C.M. et al.

**Supplementary Figure S3.** Deficiency of SMP30 results in apoptosis in human mammary carcinoma cells (A) The comparison of Bax (apoptotic marker), PCNA (cell proliferation marker), and SMP30 between MCF-7 and MDA-MB-231. (B) The bar chart for quantification of Bax expression levels in MCF-7 and MDA-MB-231 cells. (C) The bar chart for quantification of PCNA expression levels in MCF-7 and MDA-MB-231 cells. (D) Knockdown of SMP30 in MCF-7 cells. (E) Phase-contrast photomicrographs of control wells or SMP30 knockdown wells of MCF-7 cells treated with 0μM, 100μM, 200μM of H2O2. Marked cell death occurred in SMP30 knockdown wells. Scale bar = 200μm

**Supplementary Table S1. Clinical information of human breast cancer samples used for the analysis.**

| **Epidemiological data of human breast cancer used for analysis** | | | | |
| --- | --- | --- | --- | --- |
| **NO.** | **Age** | **Sex** | **Dx** | **Tumor Grade** |
| 1 | 38 | F | Invasive ductal carcinoma | 1 |
| 2 | 47 | F | Mucinous carcinoma | 1 |
| 3 | 58 | F | Invasive ductal carcinoma | 2 |
| 4 | 50 | F | Invasive ductal carcinoma | 2 |
| 5 | 43 | F | Invasive ductal carcinoma | 2 |
| 6 | 52 | F | Invasive ductal carcinoma | 2 |
| 7 | 60 | F | Invasive ductal carcinoma | 2 |
| 8 | 56 | F | Invasive ductal carcinoma | 2 |
| 9 | 43 | F | Invasive ductal carcinoma | 2 |
| 10 | 50 | F | Invasive ductal carcinoma | 2 |
| 11 | 51 | F | Invasive ductal carcinoma | 2 |
| 12 | 52 | F | Invasive ductal carcinoma | 2 |
| 13 | 53 | F | Invasive ductal carcinoma | 2 |
| 14 | 59 | F | Invasive ductal carcinoma | 2 |
| 15 | 52 | F | Invasive ductal carcinoma | 2 |
| 16 | 63 | F | Invasive ductal carcinoma | 2 |
| 17 | 65 | F | Invasive ductal carcinoma | 2 |
| 18 | 54 | F | Invasive ductal carcinoma | 2 |
| 19 | 50 | F | Invasive ductal carcinoma | 2 |
| 20 | 79 | F | Invasive ductal carcinoma | 2 |
| 21 | 38 | F | Invasive ductal carcinoma | 2 |
| 22 | 52 | F | Invasive ductal carcinoma | 2 |
| 23 | 46 | F | Invasive ductal carcinoma | 2 |
| 24 | 62 | F | Invasive ductal carcinoma | 2 |
| 25 | 69 | F | Invasive ductal carcinoma | 2 |
| 26 | 38 | F | Invasive lobular carcinoma | 2 |
| 27 | 28 | F | Medullary carcinoma | 2 |
| 28 | 70 | F | Invasive ductal carcinoma | 3 |
| 29 | 40 | F | Invasive ductal carcinoma | 3 |
| 30 | 39 | F | Invasive ductal carcinoma | 3 |
| 31 | 57 | F | Invasive ductal carcinoma | 3 |
| 32 | 40 | F | Invasive ductal carcinoma | 3 |
| 33 | 36 | F | Invasive ductal carcinoma | 3 |
| 34 | 64 | F | Invasive ductal carcinoma | 3 |
| 35 | 49 | F | Invasive ductal carcinoma | 3 |
| 36 | 41 | F | Invasive ductal carcinoma | 3 |
| 37 | 54 | F | Invasive ductal carcinoma | 3 |
| 38 | 40 | F | Invasive ductal carcinoma | 3 |
| 39 | 52 | F | Invasive ductal carcinoma | 3 |
| 40 | 38 | F | Invasive ductal carcinoma | 3 |
| 41 | 66 | F | Invasive ductal carcinoma | 3 |
| 42 | 40 | F | Invasive ductal carcinoma | 3 |
| 43 | 48 | F | Invasive ductal carcinoma | 3 |
| 44 | 49 | F | Invasive ductal carcinoma | 3 |
| 45 | 46 | F | Invasive ductal carcinoma | 3 |
| 46 | 52 | F | Invasive ductal carcinoma | 3 |
| 47 | 34 | F | Invasive ductal carcinoma | 3 |
| 48 | 30 | F | Invasive ductal carcinoma | 3 |
| 49 | 59 | F | Invasive lobular carcinoma | 3 |
| 50 | 59 | F | Invasive lobular carcinoma | 3 |
| 51 | 38 | F | Invasive lobular carcinoma | 3 |
| 52 | 48 | F | Medullary carcinoma | 3 |
| **Total N=52** | | | | |
| **Abbreviations: F; female** | | | | |

Supplementary Table S2. Signalment data from 42 dogs with mammary gland neoplasia examined.

| **Epidemiological data of canine mammary gland tumors** | | | | | |
| --- | --- | --- | --- | --- | --- |
| **Samples** | **Species** | **Breed** | **Age, y** | **Sex** | **Dx** |
| 1 | Canine | Miniature Pinscher | 8y 3m | NF | Adenoma |
| 2 | Canine | Maltese | 13y | F | Adenoma |
| 3 | Canine | Yorkshire Terrier | 13y | F | Adenoma |
| 4 | Canine | Poodle | 8y | F | Adenoma |
| 5 | Canine | Maltese | 6y | F | Adenoma |
| 6 | Canine | Shih Tzu | 13y 9m | NF | Adenoma |
| 7 | Canine | Maltese | 7y | NF | Adenoma |
| 8 | Canine | Dachshund | 10y 3m | F | Adenoma |
| 9 | Canine | Yorkshire Terrier | 18y | NF | Adenoma |
| 10 | Canine | Poodle | 6y | F | Adenoma |
| 11 | Canine | Dachshund | 10y | NF | Adenoma |
| 12 | Canine | Shih Tzu | 12y 2m | NF | Adenoma |
| 13 | Canine | Shih Tzu | 11y | F | Adenoma |
| 14 | Canine | Miniature Pinscher | 11y | NF | Adenoma |
| 15 | Canine | Maltese | 13y | F | Carcinoma |
| 16 | Canine | Maltese | 7y | F | Carcinoma |
| 17 | Canine | Poodle | 8y | F | Carcinoma |
| 18 | Canine | Maltese | 15y | F | Carcinoma |
| 19 | Canine | Mongrel | 10y | NF | Carcinoma |
| 20 | Canine | Maltese | 10y10m | F | Carcinoma |
| 21 | Canine | Dachshund | 10y3m | F | Carcinoma |
| 22 | Canine | Maltese | 8y | F | Carcinoma |
| 23 | Canine | Maltese | 13y | F | Carcinoma |
| 24 | Canine | Mongrel | 13y | F | Carcinoma |
| 25 | Canine | Maltese | Unknown | NF | Carcinoma |
| 26 | Canine | Maltese | 9y3m | F | Carcinoma |
| 27 | Canine | Cocker Spaniel | 14y | NF | Carcinoma |
| 28 | Canine | Maltese | 12y | F | Carcinoma |
| 29 | Canine | Shih Tzu | 11y 10m | NF | Carcinoma |
| 30 | Canine | Cocker Spaniel | 13y 9m | NF | Carcinoma |
| 31 | Canine | Mongrel | 6y | F | Carcinoma |
| 32 | Canine | Cocker Spaniel | 14y | F | Carcinoma |
| 33 | Canine | Siberian Husky | 14y 4m | NF | Carcinoma |
| 34 | Canine | Maltese | 7y | F | Carcinoma |
| 35 | Canine | Jin-do | 13y | F | Carcinoma |
| 36 | Canine | Pekingese | 8y | F | Carcinoma |
| 37 | Canine | Cocker Spaniel | 14y | NF | Carcinoma |
| 38 | Canine | Miniature pinscher | 6y 7m | NF | Carcinoma |
| 39 | Canine | Shih Tzu | 16y | NF | Carcinoma |
| 40 | Canine | Mongrel | 8y | NF | Carcinoma |
| 41 | Canine | Maltese | 13y3m | NF | Carcinoma |
| 42 | Canine | Poodle | 9y | F | Carcinoma |
| **Total N = 42** | | | | | |
| **Abbreviations: F, female; NF, neutered female** | | | | | |
